# Cardiac conditions in pregnancy and the role of midwives: A discussion paper

**DOI:** 10.1002/nop2.269

**Published:** 2019-04-01

**Authors:** Sandra Millington, Judith Magarey, Gustaaf A. Dekker, Robyn A. Clark

**Affiliations:** ^1^ Adelaide Nursing School The University of Adelaide Adelaide South Australia Australia; ^2^ Northern Campus Women's and Children's Division, Lyell McEwin Hospital The University of Adelaide Elizabeth Vale South Australia Australia; ^3^ School of Nursing and Midwifery Flinders University Adelaide South Australia Australia

**Keywords:** advanced midwifery education, cardiovascular diagnosis, heart disease, pregnancy complications

## Abstract

**Aim:**

This paper provides an overview of the two broad categories of cardiac conditions observed in pregnancy (congenital and acquired). It also identifies the midwives' role in the childbirth continuum and includes assessment, track and trigger systems and management during labour and delivery.

**Design:**

Discussion paper.

**Methods:**

Data were collected by reviewing international evidence and by searching computerized databases.

**Results:**

Research has identified that women with associated risk factors of a cardiac condition who delay pregnancy have an increased risk of experiencing cardiovascular complications in pregnancy with poorer outcomes. The Three Ps in a Pod clinical initiative in the United Kingdom highlights working as a team in multidisciplinary programmes to improve mothers' care and save lives. Midwives play a key role during pregnancy and need to be appraised in relation to cardiovascular disease observed in pregnancy, its potential risks and anticipated problems and within the continuum of care.

**What does this paper contribute to the wider global clinical community?**

Cardiac disease remains a major global cause of maternal mortality and morbidity. Accurate diagnosis of cardiac disease in pregnancy remains complex, since physiological changes in the cardiovascular system during pregnancy mimic disease and, therefore, present a confusing clinical picture. This paper highlights the following:

- Midwives play a key role during pregnancy and need to be appraised of the CVD observed in pregnancy, its potential risks, anticipated problems and in terms of their role within the continuum of care.
- Midwives will often be the first healthcare providers to see pregnant women with cardiac disease and can facilitate risk assessment and detection of these problems to ensure optimal management.
- There is also a potential role for midwives within the childbirth continuum to collaborate in multidisciplinary teams with ongoing surveillance; perform risk assessments; and ensure parental health education, maternal awareness and self‐management to optimize outcomes for both mother and baby.

## 1 INTRODUCTION

In 2018, the European Society of Cardiology reported a greater risk of cardiovascular disease (CVD) in pregnancy in the Western world. This was attributed to increasing age at first pregnancy and pregnancies in the late reproductive years (or between ages 40–50) that included comorbidities (Regitz‐Zagrosek et al., 2018). Historically, rheumatic heart disease (RHD) was a contributing factor for cardiac complications in pregnancy; however, today women who have undergone surgery for complex congenital heart disease (CHD) or have who have acquired cardiac disease are at high risk (Johns, 2013). This does not imply that expectant women with heart disease cannot achieve an uncomplicated pregnancy; many women do so. Good care is facilitated through appropriate preconception counselling and evaluation, when potential risks can be identified and discussed and, in some cases, through therapeutic interventions implemented to optimize the clinical status of mother and unborn child (Zöllner, Curry, & Johnson, 2013). However, accurate diagnosis of cardiac disease in pregnancy remains complex, as the physiological changes in the cardiovascular system during pregnancy mimic disease and, therefore, present a confusing clinical picture (Arafeh & Baird, 2006; Merrigan, 2009). Women with pre‐existent or newly diagnosed cardiac conditions during pregnancy require both physiological and psychological support to achieve the best outcome for both mother and baby (Revell, 2016).

The psychological state of a mother may influence transient changes in their cardiovascular system during labour and delivery, with emotions such as anxiety and fear linked to hypertension and increased vasoactive hormones (Nicholson et al., 2016; Revell, 2016). Current research has identified that perinatal depression is an important risk factor for pregnancy‐associated CVD, particularly heart failure, and contributes to poorer outcomes, in turn (Nicholson et al., 2016). These women often require complex counselling, multidisciplinary team follow‐ups and referral to a high‐risk maternity hospital. As such, midwives have an important educational and supportive role throughout a woman's pregnancy (Revell, 2016).

## 2 AIMS OF THE STUDY

The aim of this paper was to update midwives with an overview of the cardiovascular conditions observed in pregnancy, highlight management and consider their role with the health of mother and baby. Indeed, it is a challenge for midwives to reconcile a culture of risk and the changing profile of women giving birth with the philosophy of midwifery and midwifery education, as advocated by the International Confederation of Midwives (ICM) (Bharj et al., 2016). Although the phenomena of respectful care and over‐medicalization reported in Bharj et al. (2016) are acknowledged, it will not be discussed in the context of this paper. Instead, the focus is on midwives as among the first healthcare providers to see pregnant women and their role in conducting risk assessment and facilitating optimal management for women‐centred care (International Confederation of Midwives & Global Standards for Midwives Education, 2010). Research Ethics Committee approval was not required for this discussion paper.

## 3 BACKGROUND

### 3.1 The significance of cardiovascular disorders in pregnancy

Cardiac conditions typically seen in pregnancy fall into two broad categories: congenital and acquired (Johns, 2013). Corrected CHD is the most prevalent cardiac condition and is generally well tolerated, although this is dependent on the specific type of cardiac defect. Factors that influence how well a mother and her baby tolerate pregnancy include the maternal heart's functional status (i.e. the ventricular function), the severity of valvular disease and the presence of cyanosis or pulmonary hypertension (Johns, 2013). Cyanotic CHD in pregnant women also poses a significant risk for both mother and foetus, with a high incidence of stillbirth, preterm delivery and intrauterine growth restriction (IUGR) (Yu‐Ling Tan, 2010).

During 2009 and 2014 in the UK, 189 women reportedly died from heart disease associated with or aggravated by pregnancy (Knight et al., 2016). Sudden arrhythmic cardiac death with normal hearts was the most common cause of heart disease‐related deaths (*N* = 53, 35%), with ischaemia following (*N* = 34, 22%). Women who died and had pre‐existing cardiac problems comprised 17% of that sample, while 77% of women were not known to have cardiac problems. This is a reminder to be cognizant of the possibility of undiagnosed cardiac disease in pregnant or recently delivered women (Knight et al., 2016).

The same “confidential” report on acquired heart disease found that 34 women died from ischaemic‐related heart conditions, representing 22% of 153 cardiac deaths between 2009–2014 (Cantwell et al., 2011; Knight et al., 2016). Further, there were 16 deaths as a result of atherosclerosis and 11 due to coronary artery dissection (Cantwell et al., 2011; Knight et al., 2016). Increased maternal age and pre‐existent risk factors such as stress, smoking, obesity, high cholesterol, diabetes, hypertension and a family history of heart disease all contribute to the increased risk of atherosclerotic coronary artery disease (CAD) associated with death in pregnancy (Burchill et al., 2015; Cantwell et al., 2011; Johns, 2013). Acquired cardiac conditions such as acute coronary syndrome (ACS), cardiomyopathies and arrhythmias also contribute to the global cause of complications in pregnancy and between 10 and 15% of indirect maternal mortality (Babic, Gabric, & Pintaric, 2011; Cantwell et al., 2011; Elkayam et al., 2014; Smith, Young, & Greer, 2008; Turitz & Friedman, 2014; Westhoff‐Bleck, Podewski, Hilfiker, & Hilfiker‐Kleiner, 2013). RHD remains a challenge in developing countries as a major cause of maternal mortality, mostly from severe mitral stenosis, with a mortality rate of up to 5% (Yu‐Ling Tan, 2010). The latest Australian maternal data (dated between 2008–2012) reported 16 deaths from CVD, four of which were from dissection of the aorta, four were due to cardiomyopathy and two were a consequence of myocardial infarction (Humphrey et al., 2015).

With significant changes to midwifery education programmes and clinical practice in Australia (which advocate the ICM philosophy of midwifery and that pregnancy and childbirth are normal physiological processes), it is no longer routine practice for midwives to undertake a comprehensive antenatal physical examination for pregnant women (Chalmers, Mangiaterra, & Porter, 2001; Tierney, Sweet, Houston, & Ebert, 2018). However, it is beneficial to provide additional education for midwives on cardiac history, as well as assessment to address specific clinical issues for RHD for Indigenous women and recent migrants, as a component of culturally sensitive antenatal care (Bharj et al., 2016; Clarke & Boyle, 2014). The following sections detail the physiological cardiovascular adaptations during normal pregnancy, the track and trigger systems and labour and delivery information for midwives to consider in their practice.

### 3.2 Cardiovascular adaptations during normal pregnancy

The following physiological changes present unique challenges for women who may be cardiac compromised in pregnancy (Ruys, Cornette, & Roos‐Hesselink, 2013):

1. The increased intravascular volume, which peaks in the third trimester, is a potential problem in women with obstructive cardiac lesions, such as mitral or aortic stenosis, or with impaired ventricular function (Roberts & Adamson, 2013).
2. A progressive decrease in systemic vascular resistance (SVR) in pregnancy enables the mean arterial pressure (MAP) to be preserved despite an increase in cardiac output (CO). The MAP and systolic blood pressure (SBP) fall during pregnancy but returns to baseline in the late third trimester (Roberts & Adamson, 2013).
3. Marked fluctuations in CO during labour impose stress on an expectant mother's compromised cardiovascular system. During pregnancy, the overall CO increases by 30% to 50% and can unmask previously asymptomatic cardiac diseases (Roberts & Adamson, 2013). The CO then peaks in the final stage of labour from 10 to 30 min (Hall, George, & Granger, 2011; Harris, 2011; Roberts & Adamson, 2013). This increase is attributed to autotransfusion from the uterus and the venocaval release of volume in the lower extremities; however, the volume returns to the pre‐labour level within an hour of delivery. In women who have pre‐existent cardiac lesions with a fixed CO (i.e. pulmonary hypertension and mitral stenosis), adapting to these fluctuations may trigger clinical deterioration (Arafeh & Baird, 2006; Kaleschke & Baumgartner, 2011).
4. Hypercoagulability in pregnancy requires anticoagulation in women who have a high risk of arterial thrombosis and embolization or have a prosthetic heart valve. The increase in the coagulation factors VII, VIII, X and XII and fibrinogen accompanied by decreased anticoagulatory and fibrinolytic activity during pregnancy contribute to the prothrombotic state and increased risk of deep vein thrombosis. However, hypercoagulability in the peripartum period acts as a protection mechanism for major blood loss during delivery for the mother (Cox, Gogarten, & Marcus, 2005; Kaleschke & Baumgartner, 2011).

Table 1 provides a clinical reference of physiological changes that occur during pregnancy, highlighting the peak effect and potential risks.

**Table 1:** Physiological changes that occur during pregnancy, labour and delivery

Haemodynamic alterations	Time of peak effect	Potential risks
Cardiac output increased 30%–50%	20–24 weeks	Women with limited cardiac function or reserve may develop heart failure
Stroke volume increased 20%–30%	20–24 weeks	Increased preload (central venous pressure or filling pressure) presents a problem for obstructed lesions (e.g. mitral or aortic stenosis) or ventricular dysfunction
Heart rate increased 10%–20%	Third trimester	Tachycardia causes palpitations and impairs ventricular filling
Blood volume increased 40%	20–24 weeks	Physiological “anaemia” of pregnancy due to less increase in erythrocyte mass
Peripheral vasodilation due to circulating oestrogen and direct arteriovenous connection with placenta to decrease SVR	Throughout pregnancy	Decreases in both blood pressure and valvular regurgitation
Minute ventilation increased 50%	Second trimester	Sensation of tachypnoea or dyspnoea

## 4 THE ROLE OF THE MIDWIFE

### 4.1 Antenatal assessment during pregnancy

The midwives' role includes obtaining pertinent medical and cardiac history in the antepartum period. Specific risk factors such as hypertension, pre‐eclampsia, smoking, obesity, diabetes, elevated saturated fats and cholesterol that require referrals and ongoing self‐management need to be identified (Knight et al., 2016; Revell, 2016). For example, a woman with a pre‐existent cardiac state may have received preconception counselling and, thus, be well informed; however, this is not always the case. Midwives need to be appraised of the “at‐risk” populations such as Indigenous people and immigrants, particularly those who are newly arrived or from African nations.

The potential problems with new or undiagnosed cardiac dysfunction in pregnancy could be mitigated by midwives who are able to distinguish between normal and abnormal cardiovascular symptoms and initiate early referral and investigations (Roberts & Ketchell, 2012). This requires knowledge of pre‐existent cardiac conditions or conditions unmasked by the physiological changes in pregnancy (Knight et al., 2016). A recent UK‐based report emphasized that women presenting repeatedly with cardiac symptoms and objective signs such as raised respiratory rate, persistent tachycardia and orthopnoea should be fully investigated (Knight et al., 2016). Examples of the cardiovascular symptoms observed in pregnancy are summarized in Table 2. The current track and trigger systems in clinical practice for midwives can improve recognition and response to clinical deterioration during pregnancy.

**Table 2:** Cardiovascular symptoms observed in pregnancy

Due to pregnancy	Need to flag, as may represent cardiac disease
Fatigue	Palpitations, symptomatic at rest
Dizziness	
Palpitations	Persistent oedema
Lower extremity swelling/peripheral oedema	Dyspnoea, progressive, nocturnal or at rest
Dyspnoea	Orthopnoea
Nocturia	Chest pain, exertional or at rest
Chest pain	
Syncope, vasovagal	Syncope, exertional

### 4.2 Track and trigger systems

James, Endacott, and Stenhouse (2011) discussed the benefits of the current “track and trigger systems” that have been tailored to identify the altered physiology of pregnancy in the antepartum and intrapartum periods. Also known as the modified early obstetric warning system (MEOWS), this readily available tool empowers midwives to make good clinical judgements and recognize early clinical deterioration to escalate care (James et al., 2011). However, clinical effectiveness depends on accurate documentation of observations conducted at appropriate frequencies. The global trend has shifted “to examine severe maternal morbidity as well as mortality … to include women who may be moving, undetected, along the morbidity–mortality continuum” (James et al., 2011, p. 65)—and midwives are potentially ideal members of the multidisciplinary team to identify these women. However, this presents a challenge for midwives who may not have the clinical knowledge or skills to perform cardiovascular assessment. Table 3 provides a quick reference guide for midwives detailing the cardiovascular assessment findings during pregnancy that warrant further investigation.

**Table 3:** Cardiovascular physical assessment findings during pregnancy

Due to pregnancy	May represent cardiac disease; requires investigation
Tachycardia	Bradycardia (Pulse < 50 bpm)
Ectopic beats	
Dilated/distended jugular/neck veins	Tachycardia (Pulse > 150 bpm)
Bounding pulses or collapsing pulse, dynamic precordium	Jugular venous distension
Loud first heart sound	
Third heart sound	Cardiomegaly
Systolic murmur (mid‐ejection) at pulmonary area/lower left sternal edge	Right ventricular heave
Basilar rales	Loud pulmonic component of S2
Peripheral oedema	Summation gallop
	Loud systolic murmur (3–6/6)
	Diastolic murmur
	Cyanosis or clubbing
	Persistent rales
	Peripheral oedema

Knight et al. (2016) mandated ready access to an electrocardiograph (ECG) machine and someone who can interpret ECGs as priority in consultant‐led maternity units. Specialist midwives and doctors who are skilled in 12‐lead ECG interpretation should know the changes in normal pregnancy. Left‐axis deviation is considered normal, while similarly inverted or flattened T waves may be and so do not require further investigation (Canobbio et al., 2017; James et al., 2011). Pertinent findings in normal pregnancy for chest X‐ray and 12‐lead ECG investigations are outlined in Table 4. The South Australian Perinatal Practice Guidelines recommend the New York Heart Association Functional Classification Criteria tool for prediction of maternal and neonatal outcomes. These guidelines are used when counselling prospective parents to determine the level of hospital care required (SA Health, 2014). Currently, the modified World Health Organization classification is the most accurate system of risk assessment for maternal cardiovascular complications (Regitz‐Zagrosek et al., 2018).

**Table 4:** Chest X‐ray and ECG findings in normal pregnancy

Chest X‐ray	ECG
Straightened left heart border	Leftward shift of QRS axis
Increased cardiothoracic ratio	Small Q wave, inverted wave in lead III
Increased pulmonary vascular markings	ST segment and T wave changes
Small pleural effusions (early postpartum)	Atrial/ventricular ectopic

### 4.3 Labour and delivery

Midwives need to be cognizant that the haemodynamic changes during labour and delivery present potential challenges for women with heart disease; therefore, they need to anticipate and be prepared for sudden clinical deterioration. Repositioning mothers to a left lateral position to avoid compression of the inferior vena cava by the gravid uterus for women who experience supine syndrome is recommended for labour. However, this may be inappropriate for women with some specific cardiac conditions (Canobbio et al., 2017; McGregor, Barron, & Rosene‐Montella, 2015).

During each uterine contraction, a bolus of fluid is expelled into the intravascular space. Although it is transient and repetitive, for some at‐risk women, these changes may exacerbate underlying cardiac conditions. During labour, the CO increases by 15%–20% and SBP by 10% (Regitz‐Zagrosek et al., 2011). Concurrently, anxiety and pain stimulate the systemic nervous system to further elevate the SBP and heart rate (Canobbio et al., 2017; Harris, 2011). Physiological changes during labour affect various cardiac conditions differently, notably aortic diseases, obstructive valve disease and cardiomyopathy. During the second stage of labour when women use the prolonged “Valsalva” manoeuvre, increasing blood pressure and afterload can complicate aortic disease. Judicious management of fluid therapy and epidural anaesthesia may mitigate the risk of marked fluctuations in blood pressure that are detrimental to women with obstructive valve disease or cardiomyopathy (Arafeh & Baird, 2006; Roberts & Adamson, 2013).

Midwives recognize that delivery and management are tailored to a mother's specific cardiac state following a multidisciplinary team review. This team includes an obstetric high‐risk physician, a cardiologist, an anaesthetist, an obstetrician and midwives (Curtis et al., 2009; SA Health, 2014). The proposed birth plan is also clearly documented, with vaginal delivery being the preferred mode and lower (uterine) segment caesarean section remaining limited to obstetric indications (Canobbio et al., 2017; Curtis et al., 2009). In instances of maternal collapse, both resuscitation and obstetric guidelines recommend that a perimortem caesarean delivery should be carried out within four minutes, but only if there is no return of spontaneous circulation with delivery of the foetus within five minutes beyond 20 weeks' gestation (McGregor et al., 2015; Soar et al., 2010; Vanden Hoek et al., 2010). Table 5 summarizes the risks and commonly encountered problems during labour.

**Table 5:** Different diagnoses with corresponding risks and commonly encountered problems

Type of heart disease	Most common complications	Important information
Corrected CHD		
Atrial septal defect	Arrhythmias	Uncorrected ASD ↑risk of pre‐eclampsia
Ventricular septal defect	Premature delivery (12%)	Uncorrected VSD ↑risk of pre‐eclampsia
Atrioventricular septal defect	Arrhythmias (10%), deterioration of atrioventricular valve regurgitation (17%)	Reoccurrence of CHD (10%)
Tetralogy of Fallot	Arrhythmias (6%)	↑Risk of progression to right ventricular dilation secondary to severe pulmonary regurgitation
Coarctation of the aorta	Hypertensive disorders (11%)	Increased risk of aortic dissection
Transposition of the great arteries (Mustard/Senning procedures)	Arrhythmias (22%), heart failure (11%)	Irreversible ventricular dysfunction in 10%
Fontan operation	Arrhythmias (16%), heart failure (4%)	In case of cyanosis risk of miscarriage
Eisenmenger syndrome	Heart failure (21%), maternal mortality (↑50%)	Mainly in postpartum period (first 3 days)
Valvular heart disease		
Mitral stenosis	Heart failure (31%), arrhythmias (11%)	Mainly in patients with mitral valve <1.5 cm^2^
Aortic stenosis	Heart failure (3%–44%), arrhythmias (6%–25%)	Mainly in patients with an aortic valve <1.5 cm^2^
Pulmonary stenosis	Right‐sided heart failure (9%)	Mainly in patients with moderate to severe pulmonary stenosis
Regurgitation lesions	Heart failure (7%), supraventricular tachycardia (9%)	Mainly in patients with decreased cardiac function at baseline
Mechanical valves	Valvular thrombus (↑10%), maternal mortality (↑4%)	Outcome depends on anticoagulation
Cardiomyopathy		
Peripartum cardiomyopathy (current pregnancy)	Severe heart failure at the end of pregnancy (100%), maternal mortality (15%)	Complete recovery of ventricular function in half of the patients
Peripartum cardiomyopathy (previous pregnancy, minus abnormal ventricular function)	Recurrence of heart failure (21%)	Ventricular function further decreases in some patients
Peripartum cardiomyopathy (previous pregnancy, plus abnormal ventricular function)	Recurrence of heart failure (44%), maternal mortality (20%)	Ventricular function further decreases in most patients
Dilated cardiomyopathy	Heart failure (25%), arrhythmias (19%)	Mainly in patients with abnormal ventricular function (LVEF < 45%) at baseline
Hypertrophic obstructive cardiomyopathy	Heart failure (28%)	Mainly in symptomatic patients at baseline; beta‐blockers should be considered
Hypertrophic non‐obstructive Cardiomyopathy	Low risk of heart failure	Mainly in symptomatic patients at baseline
Aortic disease		
Marfan syndrome	Aortic dissection (1%–10%)	High risk in patients with aortic diameter> 45 mm
Bicuspid aortic valve disease	Aortic dissection (<1%)	High risk in patients with aortic diameter> 50 mm
Turner's syndrome	Hypertensive disorders (67%), aortic dissection (5%)	Women with Turner's syndrome often not fertile
Ehlers–Danlos syndrome	Maternal mortality (11.5%)	↑Risk of spontaneous uterine rupture
Pulmonary arterial hypertension	Maternal mortality (17%–33%)	Mainly in postpartum period (first 3 days)

Note

ASD, Atrial septal defect; LVEF, left ventricular ejection fraction; VSD, ventricular septal defect.

Pregnancy has been regarded as the ultimate cardiac stress test that a woman may experience in her life (Samuels‐Kalow & Funai, 2007; Yu‐Ling Tan, 2010). The physiological changes increase the likelihood of a complicated pregnancy and birth in women with latent cardiac dysfunction (asymptomatic cardiac dysfunction) or a pre‐existent cardiac dysfunctional state. Thus, it is important to highlight the cardiovascular adaptations that exacerbate acquired cardiac conditions.

## 5 CONGENITAL CARDIAC CONDITIONS IN PREGNANCY

### 5.1 Risks related to pregnancy and birth

The congenital defects that are likely to be problematic in pregnancy are complicated by pulmonary hypertension, cyanosis and severe left ventricular outflow tract obstruction, with cyanotic CHD posing the most significant risk to both mother and foetus (Cantwell et al., 2011; Kaleschke & Baumgartner, 2011). Cyanosis worsens during pregnancy due to the increased cardiac workload, while polycythaemia, hypoxaemia and cyanosis increase the risk of thromboembolic complications to 32% (van Mook & Peeters, 2008). Women with uncorrected cyanotic CHD also have increased cardiovascular maternal complication rates of up to 30% (Elkayam, Goland, Pieper, & Silversides, 2016), which is precisely why midwives need to be aware that pregnancy will be discouraged when mothers have an oxygen saturation of less than 85%, a haematocrit above 60% and recurrent syncope, due to maternal risk and a live birth rate of only 12% (Canobbio et al., 2017; Regitz‐Zagrosek et al., 2011). In particular, hypoxaemia is poorly tolerated by the foetus and is associated with a high incidence of foetal loss, stillbirths, preterm delivery and IUGR. In these cases, the risk to mother and unborn child correlates with the former's resting oxygen saturation (Cox et al., 2005; van Mook & Peeters, 2008; Zöllner et al., 2013).

### 5.2 Congenital valvular heart disease

Mitral valve stenosis is the most common RHD valvular lesion observed in pregnancy to require invasive intervention such as open mitral valve commissurotomy or balloon valvuloplasty prior to or during gestation (van Mook & Peeters, 2008). Prophylactic antibiotics are administered in labour to women with valve disease or a history of endocarditis or increased risk of sepsis, and the guarded use of oxytocin infusion during the third stage is recommended (Curtis et al., 2009; SA Health, 2014).

### 5.3 Pulmonary hypertension or Eisenmenger syndrome

For women with pulmonary hypertension or Eisenmenger syndrome, the increasing cardiac pressure loads in pregnancy lead to right ventricular failure during the peri‐ and postpartum period and increase the maternal mortality rate to 30%–50% (Cox et al., 2005; Kempny et al., 2017). These women require a detailed delivery plan that considers the optimal timing and mode of delivery, postpartum intensive care and mechanical support and individualized support for many months postdelivery (Regitz‐Zagrosek et al., 2018). Further, primary pulmonary artery hypertension secondary to CHD is a contraindication for pregnancy due to the poor prognosis for mother and baby, so termination is recommended (Canobbio et al., 2017; Gei & Montufar‐Rueda, 2014; Johns, 2013).

## 6 ACQUIRED CARDIAC CONDITIONS IN PREGNANCY

Acquired cardiac conditions are undetected or latent conditions where the stress of pregnancy precipitates clinical deterioration of a woman's cardiac state. In the latest UK‐based report, these conditions included ACS, aortic dissection and peripartum cardiomyopathy (PPCM; Cantwell et al., 2011; Johns, 2013; Knight et al., 2016; Ruys et al., 2013).

### 6.1 Acute coronary syndrome in pregnancy

ACS is an umbrella term that describes the spectrum of conditions resulting from a reduced coronary blood flow to the heart and range from acute myocardial ischaemia (unstable angina) to injury and necrosism, as seen in acute myocardial infarction (AMI) (Knight et al., 2016). ACS associated with the risk factors of smoking, hypertension, hyperlipidaemia, diabetes and family history in pregnancy was reported in the UK (Borna, Neamatipoor, & Radman, 2012; Clegg & Macnab, 2013; Knight et al., 2016; Pepine et al., 2015), with pregnancy‐related risk factors such as pre‐eclampsia, eclampsia, postpartum infection, thrombophilia and blood transfusion known to increase the risk of AMI between three‐ and fourfold (Cantwell et al., 2011; Elkayam et al., 2014; Ruys et al., 2013).

The causes of AMI in pregnancy are classified into two broad categories of atherosclerotic (often with cardiovascular risk factors) and non‐atherosclerotic, which includes coronary arterial dissection, thrombosis and coronary artery spasm (spontaneous or drug induced) (Wuntakal, Shetty, Ioannou, Sharma, & Kurian, 2013). Merrigan (2009) stressed the need for recommendations to be implemented across multiple nurse settings to educate and guide staff on the care of pregnant women with AMI. Women with pre‐existent CAD have an increased risk throughout pregnancy and immediately postpartum. Often, their clinical presentation will be atypical of ischaemia, with dyspnoea, vomiting or dizziness present (Cantwell et al., 2011). Although AMI in pregnancy was once considered a rare occurrence, recent statistics indicate increased incidence, with a maternal death rate ranging between 20%–37% (Knight et al., 2016; Merrigan, 2009; Wuntakal et al., 2013). With a growing number of women delaying childbirth to later reproductive years, ischaemic cardiac disease is expected to become more prevalent (Roth & Elkayam, 2008; Turitz & Friedman, 2014). Thus, women with AMI require complex care by a multidisciplinary team that includes the assistance of capable midwives (Cantwell et al., 2011; Elkayam et al., 2014; Johns, 2013; Knight et al., 2016; Merrigan, 2009).

Management of AMI is primarily interventional cardiac procedures (i.e. percutaneous coronary intervention) with introduction of bare‐metal stents and/or coronary artery bypass surgery to improve coronary blood flow (Babic et al., 2011). The aim is to avoid the use of thrombolytics until after delivery because of the increased risk of heavy haemorrhage (Knight et al., 2016; Ruys et al., 2013).

### 6.2 Aortic dissection

Aortic dissection, once considered rare in pregnancy, is now comparable to PPCM. In a recent Australian maternal mortality report, there were four deaths due to dissection of the aorta and four due to cardiomyopathy between 2008–2012 (Humphrey et al., 2015). This condition is evident in women who are diagnosed with Marfan or Ehlers–Danlos syndrome (connective tissue disorders), a bicuspid aortic valve, coarctation of the aorta (and a previous repair) or hypertension (Cantwell et al., 2011; Johns, 2013; Yu‐Ling Tan, 2010). The exact precipitating cause is uncertain, but there is speculation that pregnancy predisposes women to aortic dissection because of the hormonal changes involved and the increased haemodynamic shear stress results in structural damage to vasculature (Zöllner et al., 2013). Midwives need to be cognizant of the fact that when a pregnant woman presents with severe chest, abdominal or back pain that requires opiate analgesia (excluding labour or postoperative pain), this warrants thorough investigation (Knight et al., 2016).

### 6.3 Cardiomyopathies and heart failure

Cardiomyopathies in pregnancy include acquired and inherited diseases such as PPCM and toxic, hypertrophic, dilated, restrictive and idiopathic cardiomyopathies (Knight et al., 2016; van Mook & Peeters, 2008). Takotsubo syndrome, also known as “stress cardiomyopathy,” previously reported in postmenopausal women, is a now potential cause for acute heart failure (AHF) in birthing (Kucia, Dekker, & Arstall, 2015; Minatoguchi et al., 2014). In particular, Takotsubo syndrome is a cardiac condition that mimics ACS with no significant CAD seen on angiography, yet the characteristic ballooning of the left ventricle impairs the heart's ability to pump effectively and is evident on early echocardiogram (Brezina & Isler, 2008; Kucia, 2015; Lyon et al., 2016). Published cases describe Takotsubo syndrome as occurring soon after birthing and is associated with multiparity, preterm labour, caesarean delivery and 25% peripartum haemorrhage. It is less commonly associated with pre‐eclampsia and haemolysis, elevated liver enzymes and low platelet count syndrome (Kucia, 2015; Kucia et al., 2015). Importantly, early recognition of Takotsubo syndrome for referral to cardiology services helps ensure a full recovery is made and avoids inappropriate use of vasopressors, which have been considered counterproductive (Kucia, 2015).

### 6.4 Peripartum cardiomyopathy

Women with AHF symptoms in the last month of pregnancy or up to six months' postpartum, with the diagnosis of PPCM by exclusion, will arrive with breathlessness, orthopnoea and peripheral oedema (Johns, 2013; Knight et al., 2016). The multifactorial risk factors for PPCM are increased haemodynamic stress, advanced maternal age, multiparous pregnancies and/or multiple or teenage pregnancies, African ethnicity, smoking, diabetes, hypertension and pre‐eclampsia (Johns, 2013; Yu‐Ling Tan, 2010). Women who experience severe AHF and cardiogenic shock may require urgent delivery, mechanical cardiovascular support (i.e. use of an intra‐aortic balloon pump or left ventricular assist device) and progress to heart transplantation (Knight et al., 2016). Midwives should know that subsequent pregnancies are considered high risk, regardless of whether a woman's left ventricular function has returned to normal. Therefore, these women require care in high‐risk management facilities to address the increased risk of heart failure reoccurrence (Knight et al., 2016; van Mook & Peeters, 2008).

### 6.5 Mitral and aortic valve stenosis

Valvular disease in pregnancy is the result of RHD and the most common lesion mitral stenosis. If severe, the maternal mortality increases up to 5%, with clinical deterioration evident in the third trimester or during labour and delivery (Johns, 2013; Yu‐Ling Tan, 2010). Rheumatic mitral stenosis has an asymptomatic phase extending 10 to 20 years, so a woman's initial diagnosis may occur during pregnancy due to the cumulative effect of tachycardia, increased blood volume and cardiac output, leading to cardiac decompensation. This results in tachyarrhythmia (rapid atrial fibrillation) or acute pulmonary oedema (Yu‐Ling Tan, 2010). Approximately 10% of women with significant aortic valve stenosis suffer a cardiac event in comparison with those with mild or moderate stenosis, who experience uneventful pregnancies (Canobbio et al., 2017). Aortic valve stenosis contributes to enlargement of the left ventricle, thus restricting the coronary blood supply and causing chest pain (angina pectoris). The key message for midwives is that for women with valvular disease, the presence of a tachyarrhythmia, such as newly acquired atrial fibrillation, affects cardiac function and may cause secondary pulmonary hypertension (Canobbio et al., 2017).

### 6.6 Arrhythmias

Benign and problematic arrhythmias are observed increasingly in women with heart disease during pregnancy (Cantwell et al., 2011; Knight et al., 2016). Bradyarrhythmias are well tolerated, but some women will require insertion of a pacemaker (Ruys et al., 2013). The risk of arrhythmias peaks during labour and delivery when tachyarrhythmia (which is the most prevalent) requires prompt treatment using non‐teratogenic drug therapy or electrical therapy (Canobbio et al., 2017; van Mook & Peeters, 2008). Midwives will need to monitor closely the maternal heart rate during labour. In cases of women with pre‐existing histories of tachyarrhythmia, the risk of exacerbation is 20% to 50%, regardless of whether one has structural heart disease (Regitz‐Zagrosek et al., 2011). Sustained tachyarrhythmia is not tolerated in women with structural heart disease or CHD so they require selective drug therapy that is not toxic to the foetus (Canobbio et al., 2017). Catheter ablation therapy of the node or an accessory pathway causing the arrhythmia may be performed prior to or following pregnancy (Regitz‐Zagrosek et al., 2011). Digoxin and β‐blocking agents are first choice of drug therapy in tachyarrhythmias, and if required, electrical cardioversion can be performed safely in pregnancy (Ruys et al., 2013). The most common antiarrhythmic amiodarone is foetotoxic and, therefore, is administered as a last resort (Regitz‐Zagrosek et al., 2011).

### 6.7 Sudden arrhythmic death syndrome

According to the latest UK confidential enquiries, sudden arrhythmic death syndrome (SADS) has recently emerged as the most common cause of maternal cardiac death (38%) (Cantwell et al., 2011; Knight et al., 2016). Autopsies have shown no histopathological abnormalities of the heart to account for these women's deaths (Knight et al., 2016). SADS has been defined as “sudden death in an adult for which no cause can be identified” (Zöllner et al., 2013, p. 92) and appears to be related to arrhythmias. Associated factors to consider are cardiac hypertrophy and obesity with a body mass index between 30–45 (Regitz‐Zagrosek et al., 2011). Inherited arrhythmic disorders such as long QT syndrome increase the risk of sudden cardiac death, especially in the postpartum months; therefore, β‐blocking agents are recommended during and following pregnancy (Canobbio et al., 2017; Regitz‐Zagrosek et al., 2011). In view of increased CVD in pregnancy, we need to improve multidisciplinary collaboration, as emphasized in Knight et al. (2016). Alternatively, should we instead explore advanced practice roles such as specialist midwifery tailored to CVD? Indeed, as discussed in Multidisciplinary Management For Both Cardiac Groups7.3, midwives play a key supportive role for pregnant women with a cardiac condition in these multidisciplinary teams.

## 7 MULTIDISCIPLINARY MANAGEMENT FOR BOTH CARDIAC GROUPS

Women with cardiac disease and moderate or high risk of complication in pregnancy have better outcomes when managed by a multidisciplinary team—particularly, a pregnancy heart team (Arafeh & Baird, 2006; Regitz‐Zagrosek et al., 2018). This depends on good multidisciplinary collaboration between specialties such as congenital cardiac imaging interventional catheterization, congenital cardiac surgery and anaesthesia, heart failure, transplantation, electrophysiology and reproductive and high‐risk pregnancy services. Naturally, their individual availability may be limited for women with more complex diseases (Cusimano, Pudwell, Roddy, Cho, & Smith, 2014; Rich‐Edwards, 2012).

The teamwork for multidisciplinary management across community‐based programmes and tertiary care centres was the focus in Knight et al. (2016). This is a crucial research area, as most adults with CHD are not “cured” and require lifelong comprehensive care from specialists who have expertise in this complex arena (Rich‐Edwards, 2012). Women with previously undiagnosed heart disease during pregnancy require immediate referral to cardiologists. However, high‐risk obstetric clinics may not necessarily be supported by grown‐up congenital heart (GUCH) teams (Abdin, 2006). Hatchett, McLaren, Corrigan, and Filer (2015) reported on GUCH nursing services and found patients expressed enhanced feelings of safety in coping with the demands of pregnancy and a heart condition, as well as satisfaction with the information provided and continuity of care through important life transitions. Their research findings demonstrated the value and contribution of a specialist nursing service to women's experiences, particularly in terms of effective care coordination, monitoring and support (Hatchett et al., 2015). Given the recent reported deaths as a result of atherosclerosis and ischaemic‐related heart conditions, there also needs to be some discussion on the services required and available for women with ACS.

### 7.1 Cardiac rehabilitation programmes

Merrigan (2009) stated that women have worse outcomes than men following midlife AMI, and therefore, innovative interventions responsive to women's unique recovery need to be developed. It is recommended a woman traumatized by a cardiac event during pregnancy have a psychologist and a mental health nurse included in the multidisciplinary team involved in follow‐up care (Merrigan, 2009). Tailored cardiac rehabilitation programmes that include midwives and lactation consultants who consider the issues of women with a new baby or the appropriateness of attending cardiac rehabilitation with older, post‐AMI patients are also recommended for women with AMI during pregnancy.

“Three Ps in a Pod” is an educational initiative that improves the care of women in pregnancy. An informative poster was distributed to all women in acute medicine and emergency departments to assist clinicians in recognizing the key causes of maternal death (Knight et al., 2016). The three Ps represent “pregnancy,” “postnatal” and “pick it up” (both the phone and the problem) and help outline the key messages from the maternal mortality report published by MBRRACE‐UK (Royal College of Physicians & Surgeons of Glasgow, 2019).

This clinical resource takes the initiative to address some of the factors contributing to maternal deaths from cardiac disease, including:

1. Failure to accurately diagnose cardiac problems.
2. Lack of early involvement of senior clinicians from obstetric and cardiology multidisciplinary teams, particularly when pregnant or postpartum women present to emergency departments.
3. Underestimation of the severity of the condition.
4. Lack of communication between multidisciplinary staff.
5. Absence of clear policies addressing cardiac problems.
6. Lack of pre‐pregnancy counselling available both in paediatric cardiology transition services and to women of child‐bearing age with known cardiac disease.
7. Lack of co‐location of obstetric and cardiac services, which jeopardizes interdisciplinary working and communication (Arafeh & Baird, 2006; Canobbio et al., 2017; Knight et al., 2016).

### 7.2 Relevance to clinical practice

The Three Ps in a Pod clinical initiative in the UK highlights working as a team in multidisciplinary programmes to improve mothers' care and save lives (Knight et al., 2016). Evidently, midwives play a key role during pregnancy and need to be appraised of the CVD observed in pregnancy, including its potential risks and anticipated problems and to consider their role within the continuum of care. Midwives are well equipped to assist in the normal birthing process but require additional knowledge to address the changing needs of women with CVD. Alternatively, a specialist midwife could ensure surveillance and management that includes prompt recognition and response to clinical deterioration (James et al., 2011). Future research is needed to explore the current continuum of care for women with CVD during pregnancy to identify gaps in clinical practice.

## 8 CONCLUSION

Improving the detection of women with newly acquired cardiac conditions, and improving surveillance and maintaining the continuum of cardiac care for women with CHD will together improve the outcome for both mother and baby. As shown, the role of midwives in childbirth facilitates ongoing surveillance, risk assessment, parental health education, maternal awareness and self‐management to optimize outcomes.

## CONFLICT OF INTEREST

The authors of this paper have no conflicts of interest in conducting this study. The authors declare that this study was conducted in the absence of any financial or commercial relationships that could be construed as a potential conflict of interest.
